# Bis(2-hy­droxy­imino­methyl-6-meth­oxy­phenolato-κ^2^ *N*,*O* ^1^)copper(II)

**DOI:** 10.1107/S1600536812032187

**Published:** 2012-08-11

**Authors:** Svitlana R. Petrusenko, Yaroslava I. Belozub, Volodymyr N. Kokozay, Irina V. Omelchenko

**Affiliations:** a Department of Inorganic Chemistry, Taras Shevchenko National University of Kyiv, 64 Volodymyrs’ka St, Kyiv 01601, Ukraine; b STC ‘Institute for Single Crystals’, National Academy of Sciences of Ukraine, 60 Lenina Ave, Kharkiv 61001, Ukraine

## Abstract

In the title compound, [Cu(C_8_H_8_NO_3_)_2_], the nearly planar mol­ecule (r.m.s. deviation = 0.037 Å) is centrosymmetric with the Cu^II^ atom lying on an inversion center. The Cu^II^ atom is tetra­coordinated, displaying a slightly distorted square-planar geometry. The main deviation from the ideal geometry is seen in the differences in the Cu—O [1.8833 (10) Å] and Cu—N [1.9405 (13) Å] bond lengths, while angular deviations are less than 3°. Intra­molecular O—H⋯O and inter­molecular C*sp^2^*—H⋯O hydrogen bonds form *S*(5) and *R*_2_^2^(8) ring motifs, respectively. The latter inter­action results in chains of mol­ecules along [100].

## Related literature

For related structures, see: Zhang *et al.* (2008 ▶); Li *et al.* (2004 ▶), 2009 ▶). For bond-valence-sum calculations, see: Brown & Altermatt (1985 ▶). For *in situ* formation of polydentate ligands, see: Coxall *et al.* (2000 ▶). For background to direct synthesis, see: Makhankova (2011 ▶).
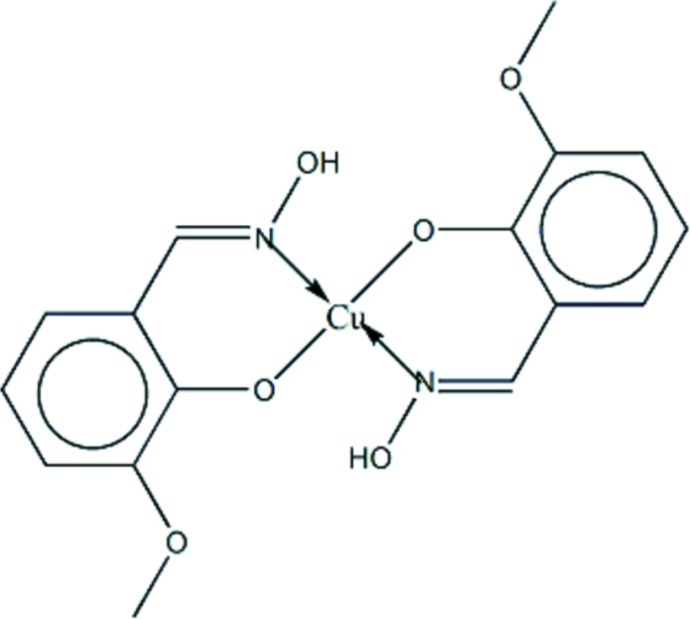


## Experimental

### 

#### Crystal data

[Cu(C_8_H_8_NO_3_)_2_]
*M* *_r_* = 395.85
Monoclinic, 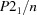

*a* = 8.4906 (4) Å
*b* = 4.8997 (2) Å
*c* = 18.9309 (9) Å
β = 94.906 (4)°
*V* = 784.67 (6) Å^3^
*Z* = 2
Mo *K*α radiation
μ = 1.43 mm^−1^
*T* = 293 K
0.50 × 0.20 × 0.10 mm

#### Data collection

Oxford Diffraction Xcalibur/Sapphire3 diffractometer
Absorption correction: multi-scan (*CrysAlis RED*; Oxford Diffraction, 2010 ▶) *T*_min_ = 0.535, *T*_max_ = 0.763
8497 measured reflections
2245 independent reflections
1816 reflections with *I* > 2σ(*I*)
*R*_int_ = 0.020

#### Refinement

*R*[*F* ^2^ > 2σ(*F* ^2^)] = 0.028
*wR*(*F* ^2^) = 0.077
*S* = 1.01
2245 reflections
132 parameters
H atoms treated by a mixture of independent and constrained refinement
Δρ_max_ = 0.39 e Å^−3^
Δρ_min_ = −0.19 e Å^−3^

### 

Data collection: *CrysAlis CCD* (Oxford Diffraction, 2010 ▶); cell refinement: *CrysAlis CCD*; data reduction: *CrysAlis RED* (Oxford Diffraction, 2010 ▶); program(s) used to solve structure: *SHELXTL* (Sheldrick, 2008 ▶); program(s) used to refine structure: *SHELXTL*; molecular graphics: *SHELXTL*; software used to prepare material for publication: *publCIF* (Westrip, 2010 ▶).

## Supplementary Material

Crystal structure: contains datablock(s) I, global. DOI: 10.1107/S1600536812032187/lr2071sup1.cif

Structure factors: contains datablock(s) I. DOI: 10.1107/S1600536812032187/lr2071Isup2.hkl

Additional supplementary materials:  crystallographic information; 3D view; checkCIF report

## Figures and Tables

**Table 1:** Hydrogen-bond geometry (Å, °)

*D*—H⋯*A*	*D*—H	H⋯*A*	*D*⋯*A*	*D*—H⋯*A*
O3—H3*O*⋯O1	0.82	1.94	2.5840 (16)	134
C7—H7⋯O3^i^	0.903 (19)	2.49 (2)	3.3231 (19)	154.3 (15)

Symmetry code: (i) 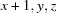.

**Fig. 1.**
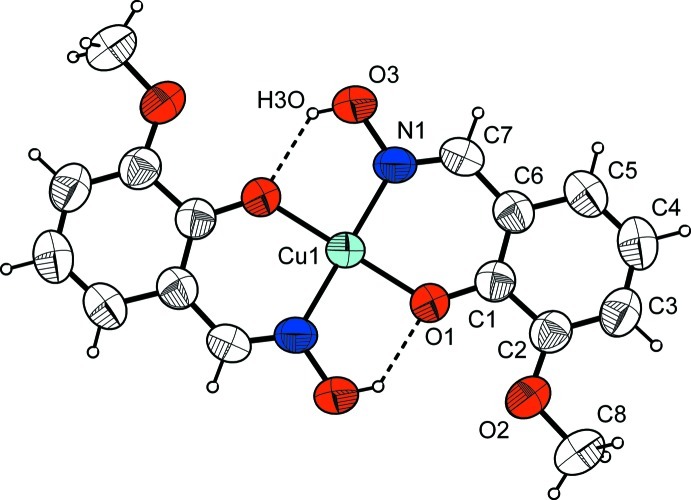
Molecular structure of [Cu(HL)2] with 70% probability displacement elipsoids for non-H atoms. The dashed lines denote hydrogen bonds.

**Fig. 2.**
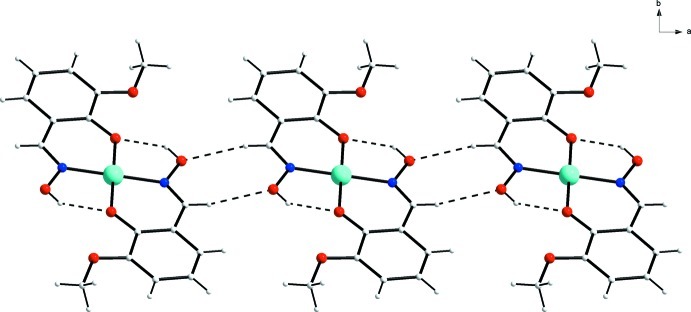
Fragment of chain-like alignment of [Cu(HL)2] viewed along the [100] direction.

## supplementary crystallographic information

### Comment

Aiming to prepare Cu/Mn heterometallic complexes with an ONO donor Shiff base (H_2_L = 2-hydroxyiminomethyl-6-methoxyphenol) the following system based on "direct synthesis" methodology (Makhankova, 2011) has been investigated: Cu^0^ – Mn^0^ – *o*-vianillin– NH_2_OH^.^HCl – NH_4_*X* – solv (in open air), where *o*-vainillin = 2-hydroxy-3-methoxybenzaldehyde; *X* = Cl, Br, I; solv = CH_3_OH, dymethylformamide(dmf), dymethylsulfoxide(dmso).

In all cases the total dissolution of copper and manganese powders was observed within 5 - 6 h resulting into intensive dark green solutions. X-ray quality crystalls were obtained from the systems with NH_4_Br in dmso and NH_4_Cl in dmf, but in the former one the yield was some better.

The asymmetric unit of [Cu(HL)_2_] includes one-half of the molecule with Cu atom occupying the (1/2 1/2 1/2) special position of multiplicity 2. The coordination geometry of the metal atom is square-planar, with the CuN_2_O_2_ chromophore, formed by means of two imine nitrogen atoms and two phenolate oxygen atoms of the two monodeptotonated Schiff base ligands realising their bidentate chelate function, [1.1_1_1_1_0] by Harris notation (Coxall *et al.*, 2000) (Fig. 1). Difference between Cu–O and Cu–N bond lengths (Table 1) causes significant linear distortion of the square. Deviations in bond angles at the Cu atom are less than 3°. The bond valence sum analysis applied to the appropriate bond lengths supports the +2 oxidation state for copper, BVS(Cu) = 2.003 (Brown & Altermatt, 1985).

Hydrogen bonds(HBs) play the principal role in the crystal structure of [Cu(HL)_2_]. The N–OH group takes part in simultaneous formation of a strong intramolecular O(3)–H(3O)···O(1) hydrogen bond with O_phenolate_ of the second ligand and a weak *inter*-molecular C(7)–H(7)···O(3)' hydrogen bond with C(*sp*^2^)–H group of the neighboring molecule (Fig. 2, Table 2). As a result, two ligands being coordinated to the Cu^II^ ion form some analogue of a macrocyclic ligand [R14] based on HBs which binds to the copper center generating two 6-membered (with only covalent bonds) and two 5-membered (with covalent and hydrogen bonds) rings (Fig. 1). It is worth noting that all known structures with H_2_L, namely Co(HL)_2_ (Zhang *et al.*, 2008), Ni(HL)_2_ (Li *et al.*, 2009) and VO(HL)_2_ (Li *et al.*, 2004), are built in the same manner demonstrating high thermodynamic stability of such structure.

The adjacent molecules join through complementary C–H···O HBs, [*R*_2_^2^(8)] synthon, forming one-dimentional stair-like ribbons along (100) direction (Fig. 2).

### Experimental

Copper powder (0.06 g, 1 mmol), manganese powder (0.05 g, 1 mmol), *o*-vainillin (0.46 g, 3 mmol), hydroxylamine hydrochloride (0.21 g, mmol) and NH_4_Br (0.20 g, 2 mmol) were added to 10 ml of dimethylsulfoxide. The mixture was stirred magnetically at 323 – 333 K until total dissolution of metal powders was observed (*ca*5 h). Goldish-green needle crystals that precipitated after 1 day, were collected by filtration, washed with methanol and dried in air; yield 32% based on Cu. IR(KBr, cm^-1^): 3080(*m*), 3057(*m*), 3006(*m*), 2959(*m*), 2936(*m*), 2834(*m*), 1649(*m*), 1598(*m*), 1554(w), 1510(*m*), 1468(*s*), 1451(*s*), 1353(w), 1332(*m*), 1302(*s*), 1247(*s*), 1216(*s*), 1195(*m*), 1101(*m*), 1081(*m*), 1018(*m*), 969(*s*), 932(w), 863(*m*), 778(*m*), 755(*m*), 735(*s*), 712(*s*), 624(*m*), 575(w), 546(w), 502(w).

### Refinement

Structure was solved by direct method and refined against F^2^ within anisotropic approximation for all non-hydrogen atoms. All hydrogen atoms were located from difference Fourier map and refined isotropically, except phenyl (H(3) - H(5)) and hydroxyl (H(3O)) H atoms that were allowed to ride on their attached atoms with C—H = 0.93 (1) Å and *U*_iso_(H)= 1.2Ueq(C) for phenyl, and C—H = 0.82 (1) Å and *U*_iso_(H)= 1.5Ueq(C) for hydroxyl H atoms. Coordinates of Cu(1) were constrained to special position (*x*=0.5000, *y*=0.5000, *z*=0.5000).

### Crystal data

**Table:**

[Cu(C_8_H_8_NO_3_)_2_]	*F*(000) = 406
*M**_r_* = 395.85	*D*_x_ = 1.675 Mg m^−^^3^
Monoclinic, *P*2_1_/*n*	Mo *K*α radiation, λ = 0.71073 Å
Hall symbol: -P 2yn	Cell parameters from 2721 reflections
*a* = 8.4906 (4) Å	θ = 3.1–32.2°
*b* = 4.8997 (2) Å	µ = 1.43 mm^−^^1^
*c* = 18.9309 (9) Å	*T* = 293 K
β = 94.906 (4)°	Needle, gold–green
*V* = 784.67 (6) Å^3^	0.50 × 0.20 × 0.10 mm
*Z* = 2	

### Data collection

**Table:**

Oxford Diffraction Xcalibur/Sapphire3 diffractometer	2245 independent reflections
Radiation source: Enhance (Mo) X-ray Source	1816 reflections with *I* > 2σ(*I*)
Graphite monochromator	*R*_int_ = 0.020
Detector resolution: 16.1827 pixels mm^-1^	θ_max_ = 30.0°, θ_min_ = 3.9°
ω scans	*h* = −11→11
Absorption correction: multi-scan (*CrysAlis RED*; Oxford Diffraction, 2010)	*k* = −6→6
*T*_min_ = 0.535, *T*_max_ = 0.763	*l* = −26→26
8497 measured reflections	

### Refinement

**Table:**

Refinement on *F*^2^	Primary atom site location: structure-invariant direct methods
Least-squares matrix: full	Secondary atom site location: difference Fourier map
*R*[*F*^2^ > 2σ(*F*^2^)] = 0.028	Hydrogen site location: difference Fourier map
*wR*(*F*^2^) = 0.077	H atoms treated by a mixture of independent and constrained refinement
*S* = 1.01	*w* = 1/[σ^2^(*F*_o_^2^) + (0.0486*P*)^2^] where *P* = (*F*_o_^2^ + 2*F*_c_^2^)/3
2245 reflections	(Δ/σ)_max_ = 0.001
132 parameters	Δρ_max_ = 0.39 e Å^−^^3^
0 restraints	Δρ_min_ = −0.19 e Å^−^^3^
11 constraints	

### Special details

**Table:**

Experimental. CrysAlis RED, Oxford Diffraction Ltd., 2010. Empirical absorption correction using spherical harmonics, implemented in SCALE3 ABSPACK scaling algorithm.
Geometry. All e.s.d.'s (except the e.s.d. in the dihedral angle between two l.s. planes) are estimated using the full covariance matrix. The cell e.s.d.'s are taken into account individually in the estimation of e.s.d.'s in distances, angles and torsion angles; correlations between e.s.d.'s in cell parameters are only used when they are defined by crystal symmetry. An approximate (isotropic) treatment of cell e.s.d.'s is used for estimating e.s.d.'s involving l.s. planes.
Refinement. Refinement of *F*^2^ against ALL reflections. The weighted *R*-factor *wR* and goodness of fit *S* are based on *F*^2^, conventional *R*-factors *R* are based on *F*, with *F* set to zero for negative *F*^2^. The threshold expression of *F*^2^ > σ(*F*^2^) is used only for calculating *R*-factors(gt) *etc*. and is not relevant to the choice of reflections for refinement. *R*-factors based on *F*^2^ are statistically about twice as large as those based on *F*, and *R*- factors based on ALL data will be even larger.

### Fractional atomic coordinates and isotropic or equivalent isotropic displacement parameters (Å^2^)

**Table:**

	*x*	*y*	*z*	*U*_iso_*/*U*_eq_	
Cu1	0.5000	0.5000	0.5000	0.03633 (10)	
N1	0.27576 (15)	0.5566 (3)	0.50728 (7)	0.0398 (3)	
C1	0.60680 (18)	0.0698 (3)	0.59715 (8)	0.0378 (3)	
O1	0.49066 (12)	0.2223 (2)	0.56829 (5)	0.0446 (2)	
O2	0.41409 (14)	−0.1275 (2)	0.66227 (6)	0.0541 (3)	
C2	0.56941 (19)	−0.1252 (3)	0.64921 (7)	0.0416 (3)	
O3	0.20159 (13)	0.3924 (3)	0.55453 (6)	0.0518 (3)	
H3O	0.2665	0.2879	0.5745	0.078*	
C3	0.6860 (2)	−0.2899 (3)	0.68168 (8)	0.0488 (4)	
H3	0.6603	−0.4164	0.7155	0.059*	
C4	0.8415 (2)	−0.2687 (3)	0.66435 (8)	0.0512 (4)	
H4	0.9189	−0.3814	0.6865	0.061*	
C5	0.8809 (2)	−0.0838 (4)	0.61520 (9)	0.0467 (3)	
H5	0.9853	−0.0700	0.6043	0.056*	
C6	0.76430 (18)	0.0881 (3)	0.58031 (8)	0.0393 (3)	
C7	0.81554 (18)	0.2753 (3)	0.52815 (8)	0.0420 (3)	
H7	0.920 (2)	0.279 (4)	0.5214 (9)	0.054 (5)*	
C8	0.3647 (3)	−0.3339 (4)	0.70831 (10)	0.0572 (4)	
H8A	0.392 (3)	−0.514 (4)	0.6925 (12)	0.050 (6)*	
H8B	0.418 (3)	−0.314 (4)	0.7595 (12)	0.072 (6)*	
H8C	0.255 (3)	−0.322 (4)	0.7053 (10)	0.064 (6)*	

### Atomic displacement parameters (Å^2^)

**Table:**

	*U*^11^	*U*^22^	*U*^33^	*U*^12^	*U*^13^	*U*^23^
Cu1	0.03102 (14)	0.03680 (14)	0.04131 (15)	−0.00245 (9)	0.00388 (9)	0.00387 (9)
N1	0.0332 (6)	0.0452 (6)	0.0413 (6)	−0.0056 (5)	0.0060 (5)	0.0017 (5)
C1	0.0405 (7)	0.0349 (6)	0.0379 (7)	−0.0014 (6)	0.0017 (6)	−0.0020 (5)
O1	0.0358 (5)	0.0457 (5)	0.0525 (5)	0.0010 (4)	0.0052 (4)	0.0134 (5)
O2	0.0521 (7)	0.0494 (6)	0.0621 (7)	−0.0034 (5)	0.0118 (5)	0.0163 (6)
C2	0.0463 (8)	0.0360 (7)	0.0422 (7)	−0.0041 (6)	0.0021 (6)	−0.0008 (6)
O3	0.0370 (5)	0.0640 (7)	0.0550 (6)	−0.0058 (6)	0.0083 (5)	0.0176 (6)
C3	0.0611 (10)	0.0393 (7)	0.0451 (7)	−0.0020 (7)	−0.0013 (7)	0.0044 (6)
C4	0.0554 (9)	0.0464 (8)	0.0499 (8)	0.0099 (7)	−0.0072 (7)	−0.0004 (7)
C5	0.0402 (8)	0.0483 (7)	0.0505 (8)	0.0060 (7)	−0.0024 (7)	−0.0055 (7)
C6	0.0391 (7)	0.0369 (6)	0.0410 (7)	0.0000 (6)	−0.0009 (6)	−0.0049 (6)
C7	0.0320 (7)	0.0477 (8)	0.0463 (7)	−0.0020 (6)	0.0033 (6)	−0.0023 (6)
C8	0.0656 (12)	0.0517 (9)	0.0560 (10)	−0.0083 (9)	0.0151 (9)	0.0089 (8)

### Geometric parameters (Å, º)

**Table:**

Cu1—O1^i^	1.8833 (10)	C3—C4	1.392 (3)
Cu1—O1	1.8833 (10)	C3—H3	0.9300
Cu1—N1	1.9405 (13)	C4—C5	1.361 (2)
Cu1—N1^i^	1.9405 (13)	C4—H4	0.9300
N1—C7^i^	1.281 (2)	C5—C6	1.419 (2)
N1—O3	1.3928 (17)	C5—H5	0.9300
C1—O1	1.3179 (17)	C6—C7	1.442 (2)
C1—C6	1.404 (2)	C7—N1^i^	1.281 (2)
C1—C2	1.428 (2)	C7—H7	0.903 (19)
O2—C2	1.362 (2)	C8—H8A	0.965 (18)
O2—C8	1.422 (2)	C8—H8B	1.04 (2)
C2—C3	1.380 (2)	C8—H8C	0.93 (2)
O3—H3O	0.8200		
			
O1^i^—Cu1—O1	180.0	C4—C3—H3	119.7
O1^i^—Cu1—N1	92.56 (5)	C5—C4—C3	120.27 (15)
O1—Cu1—N1	87.44 (5)	C5—C4—H4	119.9
O1^i^—Cu1—N1^i^	87.44 (5)	C3—C4—H4	119.9
O1—Cu1—N1^i^	92.56 (5)	C4—C5—C6	120.76 (16)
N1—Cu1—N1^i^	180.00 (8)	C4—C5—H5	119.6
C7^i^—N1—O3	114.91 (13)	C6—C5—H5	119.6
C7^i^—N1—Cu1	127.46 (11)	C1—C6—C5	119.77 (15)
O3—N1—Cu1	117.60 (10)	C1—C6—C7	123.03 (14)
O1—C1—C6	124.28 (14)	C5—C6—C7	117.20 (15)
O1—C1—C2	117.57 (14)	N1^i^—C7—C6	124.28 (14)
C6—C1—C2	118.15 (14)	N1^i^—C7—H7	117.8 (12)
C1—O1—Cu1	128.33 (10)	C6—C7—H7	117.8 (12)
C2—O2—C8	117.25 (14)	O2—C8—H8A	111.8 (14)
O2—C2—C3	125.65 (14)	O2—C8—H8B	112.2 (12)
O2—C2—C1	113.99 (13)	H8A—C8—H8B	106.2 (18)
C3—C2—C1	120.35 (15)	O2—C8—H8C	105.2 (13)
N1—O3—H3O	109.5	H8A—C8—H8C	107.6 (19)
C2—C3—C4	120.69 (15)	H8B—C8—H8C	113.9 (17)
C2—C3—H3	119.7		
			
O1^i^—Cu1—N1—C7^i^	2.42 (14)	C6—C1—C2—C3	0.0 (2)
O1—Cu1—N1—C7^i^	−177.58 (14)	O2—C2—C3—C4	−179.65 (14)
O1^i^—Cu1—N1—O3	−179.73 (11)	C1—C2—C3—C4	0.1 (2)
O1—Cu1—N1—O3	0.27 (11)	C2—C3—C4—C5	0.3 (2)
C6—C1—O1—Cu1	−0.8 (2)	C3—C4—C5—C6	−0.6 (2)
C2—C1—O1—Cu1	178.82 (10)	O1—C1—C6—C5	179.24 (14)
N1—Cu1—O1—C1	−177.96 (12)	C2—C1—C6—C5	−0.4 (2)
N1^i^—Cu1—O1—C1	2.04 (12)	O1—C1—C6—C7	−1.0 (2)
C8—O2—C2—C3	−6.2 (2)	C2—C1—C6—C7	179.38 (13)
C8—O2—C2—C1	174.03 (14)	C4—C5—C6—C1	0.7 (2)
O1—C1—C2—O2	0.10 (19)	C4—C5—C6—C7	−179.06 (14)
C6—C1—C2—O2	179.74 (13)	C1—C6—C7—N1^i^	0.6 (2)
O1—C1—C2—C3	−179.64 (13)	C5—C6—C7—N1^i^	−179.65 (15)

Symmetry code: (i) −*x*+1, −*y*+1, −*z*+1.

### Hydrogen-bond geometry (Å, º)

**Table:**

*D*—H···*A*	*D*—H	H···*A*	*D*···*A*	*D*—H···*A*
O3—H3*O*···O1	0.82	1.94	2.5840 (16)	134
C7—H7···O3^ii^	0.903 (19)	2.49 (2)	3.3231 (19)	154.3 (15)

Symmetry code: (ii) *x*+1, *y*, *z*.
